# Genetics of adaptation: Experimental test of a biotic mechanism driving divergence in traits and genes

**DOI:** 10.1002/evl3.135

**Published:** 2019-08-26

**Authors:** Diana J. Rennison, Seth M. Rudman, Dolph Schluter

**Affiliations:** ^1^ Department of Zoology and Biodiversity Research Centre University of British Columbia Vancouver British Columbia Canada; ^2^ Institute of Ecology and Evolution University of Bern Bern Switzerland; ^3^ Department of Biology University of Pennsylvania Philadelphia Pennsylvania 19104

**Keywords:** Adaptation, diversification, ecological genetics, experimental evolution, natural selection, threespine stickleback

## Abstract

The genes underlying adaptations are becoming known, yet the causes of selection on genes—a key step in the study of the genetics of adaptation—remains uncertain. We address this issue experimentally in a threespine stickleback species pair showing exaggerated divergence in bony defensive armor in association with competition‐driven character displacement. We used semi‐natural ponds to test the role of a native predator in causing divergent evolution of armor and two known underlying genes. Predator presence/absence altered selection on dorsal spines and allele frequencies at the *Msx2a* gene across a generation. Evolutionary trajectories of alleles at a second gene, *Pitx1*, and the pelvic spine trait it controls, were more variable. Our experiment demonstrates how manipulation of putative selective agents helps to identify causes of evolutionary divergence at key genes, rule out phenotypic plasticity as a sole determinant of phenotypic differences, and eliminate reliance on fitness surrogates. Divergence of predation regimes in sympatric stickleback is associated with coevolution in response to resource competition, implying a cascade of biotic interactions driving species divergence. We suggest that as divergence proceeds, an increasing number of biotic interactions generate divergent selection, causing more evolution in turn. In this way, biotic adaptation perpetuates species divergence through time during adaptive radiation in an expanding number of traits and genes.

**Impact summary**

The genes underlying the evolution of differences between species are quickly being identified in many species, but the causes of natural selection on these genes are largely unknown. We manipulated the presence of a native predator to test the effect of contrasting predation regimes on the evolution of defensive armor and at two key genes underlying armor variation between two coexisting stickleback species. The predator altered the pattern of natural selection on armor and on two underlying loci, leading to divergent evolutionary trajectories in the next generation. The study shows how direct manipulation can yield insights into the mechanisms of evolution, in this case the role of a biotic interaction. Beyond illuminating the relationships between natural selection on phenotype and genotype this experiment also demonstrates how evolution in habitat use, driven by competition, can lead to changes in the strength of other species interactions that ultimately drive further divergence. This is an empirical example of how trophic complexity can facilitate diversification and suggests that diverse and evolving biotic interactions could be a core component that sustains species divergence and speciation in adaptive radiations.

The genes underlying evolution of differences between species have been identified in many cases, but the causes of natural selection on genes and resulting phenotypes are little known (Barrett and Hoekstra 2011; Nosil 2012). A key challenge in determining the selective agents shaping genetic and phenotypic differences lies in disentangling the contribution of particular ecological factors in natural populations. We address the problem experimentally, focusing on a biotic cause of divergence at two genes underlying differences in bony defensive spines between sympatric stickleback species. In one of the species, a deletion of an enhancer of the *Pitx1* locus confers loss of the pelvic spines and girdle (Chan et al. 2010), and reduced dorsal spine length results from a splicing variant of the *Msx2a* gene (Howes et al. 2017). We test the hypothesis that interactions between the two coevolving stickleback species and a vertebrate predator have led to divergence in these armor traits and genes. We disentangle the effect of the predator from other causes by manipulating its presence/absence, rather than by introducing the prey species between locales that may differ in multiple environmental features. We carry out the experiment at a spatial scale sufficient to allow natural avoidance behaviors by prey to affect the outcome, and we use changes at the genes and phenotypes to measure evolution across a generation.

Pairs of threespine stickleback consisting of a benthic and a limnetic form (Fig. 1) provide an ideal system in which to examine the role of predation and other biotic interactions in divergence. Sympatric benthic and limnetic pairs have evolved independently several times within the last 12,000 years (Taylor and McPhail 1999) and have repeatedly diverged in many traits (Schluter and McPhail 1992). Observational studies and within‐generation selection experiments show that ecological character displacement driven by resource competition has led to the evolution of differences between sympatric species in numerous morphological traits that increase feeding performance on habitat‐specific prey types (Schluter and McPhail 1992; Schluter 1994; Schluter 2003). Single‐species (“solitary”) stickleback populations occurring in otherwise similar lakes are intermediate in trophic traits and have a generalist diet (Schluter and McPhail 1992). At the same time, patterns of divergence in traits not directly related to feeding suggest involvement of a broader suite of ecological interactions in the divergence of sympatric species (Vamosi and Schluter 2004). For example, compared to solitary stickleback populations, benthic‐limnetic pairs repeatedly show exaggerated divergence in the length of bony spines and other armor defenses against vertebrate predators (cutthroat trout, *Oncorhynchus clarkii clarkii*, and piscivorous diving birds; Reimchen 1980; Vamosi and Schluter 2002; Vamosi and Schluter 2004). Vertebrate predators preferentially exploit the open water habitat utilized by the more armored limnetic species, whereas the armor‐reduced benthic species utilizes the vegetated littoral zone of lakes where insect predators are more common (Vamosi and Schluter 2002). However, the native lakes are small, the two habitats are adjacent throughout, and individual stickleback can move freely between them.

**Figure 1:**
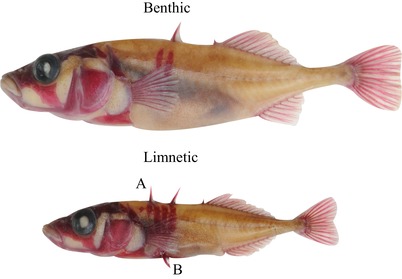
Benthic and limnetic stickleback ecotypes from Paxton Lake. Fish specimens are stained with Alizarin red to highlight bone. The letter A indicates first dorsal spine and B indicates pelvic spine; both traits are most often absent in benthic fish.

We tested whether divergence of armor between sympatric stickleback is driven by their interactions with the trout predator, an interaction that evolved in conjunction with ecological character displacement and a corresponding shift in habitat use. To maximize variation in traits and underlying genes, and yield a sensitive measure of selection and evolution, we used second generation hybrids between benthic and limnetic stickleback as our target experimental population. Although ponds are not the same as lakes, they are otherwise unmanipulated water bodies that, as we show, are sufficiently large to permit natural behaviors to mediate outcomes of natural selection (for example, differential resource use; Arnegard et al. 2014). We estimated phenotypes and genotypes for the F_2_ generation before addition of trout and tracked phenotype and allele frequencies into the F_3_ generation after one year of differential selection.

## Methods

### COLLECTION OF EXPERIMENTAL FISH

The experimental fish were the product of four F_1_ crosses made in the spring of 2011, between four pairs of benthic mothers and limnetic fathers collected from Paxton Lake on Texada Island, British Columbia, Canada. We used hybrids as the target populations in our experiment, to maximize variation for selection to act upon and to generate segregation of traits and alleles from the separate species. The range of phenotypes observed in each benthic‐limnetic F_2_ cross encompassed the variation found between the benthic and limnetic ecotypes; some F_2_ offspring lacked the first dorsal and/or pelvic spines (the benthic phenotype) others had long spines (the limnetic phenotype), with many individuals possessing intermediate spine length values. The F_0_ benthic and limnetic fish possessed the typical armor phenotypes of their ecotype: all four benthic mothers lacked pelvic spines and three of the four lacked first dorsal spines (the fourth had a short first dorsal spine), the limnetic fathers all had pelvic spines and first dorsal spines.

### THE EXPERIMENTAL PONDS

The experiment was conducted in eight semi‐natural experimental ponds located on the University of British Columbia Campus in Vancouver, Canada. The ponds were constructed in 2008 and are 25 m × 15 m, encompassing both a vegetated littoral zone and a 6 m deep open water habitat. The ponds contain a natural assemblage of food resources and do not exclude invertebrate or avian predators. For further details of the pond structure see Arnegard et al. 2014 and Figure S1 for an aerial photo.

### EXPERIMENTAL FISH AND POND INTRODUCTIONS

The experiment was conducted in four pairs of ponds (see Fig. S2 for schematic of experimental design). Pairing was based on similarity of environments according to count surveys of macrophyte coverage, phytoplankton, zooplankton, and insects. The F_1_ hybrids were reared in the laboratory in 100 L tanks for a year prior to their introduction into the experimental ponds in May 2012. Each of the four F_1_ families was split between a pair of ponds, with one cross per pond pair. Each pond received 21–31 individuals, with paired ponds receiving equal numbers of fish. The F_1_ hybrid stickleback in all eight ponds reproduced naturally over the spring and summer of 2012, producing the first pond generation composed of multiple F_2_ hybrid families.

### POND SAMPLING

In September 2012, a lethal sample of F_2_ offspring was taken from each pond. After this initial sampling was complete two coastal cutthroat trout (10–12 inches in length) were introduced to one randomly chosen pond within each pond pair (hereafter referred to as “trout addition ponds”). Cutthroat trout were obtained by angling in Placid Lake, southwestern British Columbia. The F_2_ generation was again lethally sampled in January 2013 and April 2013. In the spring and summer of 2013, the F_2_ generation fish bred within the ponds creating the F_3_ generation. This F_3_ generation was lethally sampled in September 2013. During all sampling periods, sticklebacks were caught using a combination of un‐baited minnow traps, open water seining, and dip netting. We then sub‐sampled randomly from all captured individuals. Trout did not breed within the ponds. See Figure S2 for a schematic of the experimental design and sampling timeline. Across timepoints and treatments, the estimated average population density of stickleback (indicated from mark recapture data) ranged from 693 to 1977 (Rudman et al. 2016), so the sampling of 50 individuals constituted a subsample of between 2% and 7% of the estimated total population.

### PHENOTYPING

Immediately following collection, fish were euthanized in MS‐222 and placed in 95% ethanol. A portion of the caudal fin was removed and set aside for DNA extraction. Each fish was then stained with alizarin red to highlight bony structures (Peichel et al. 2001) and the length of its first dorsal spine, pelvic spine, and standard length were measured then size corrected (see online supplement for full details). All analyses reported in this paper were undertaken using these size corrected measurements. A total of 50 individuals per pond were measured in September 2012, January 2013, April 2013, and September 2013.

### GENOTYPING, LINKAGE, AND QUANTITATIVE TRAIT LOCUS MAPPING

DNA was extracted from each fish's fin clip using a standard phenol‐chloroform extraction protocol. Fifty individuals were sampled per pond from September 2012 F_2_s and September 2013 F_3_s (800 individuals total). DNA was also extracted from the F_1_ parents and pure benthic or limnetic grandparental individuals. DNA was prepared for Illumina sequencing using the *PstI* enzyme following the genotyping by sequencing method of Elshire et al. 2011 (see Supporting Information for full details). Sequence variants were identified using a standard, reference‐based bioinformatics pipeline (see archived code and online supplement for full details). A pedigree was constructed using the MasterBayes R package (Hadfield 2012) and JoinMap (Ooijen and Voorrips 2002) was used to estimate the genetic map (see online supplement for full details). A total of 2243 SNP markers and the genetic map were used for the quantitative trait locus (QTL) mapping of first dorsal spine and pelvic spine length. QTL mapping was done using the Haley–Knott regression with F_1_ family as a covariate in the R/qtl package (Broman and Wu 2013) (see Supporting Information for full details).

### SELECTION ANALYSES

We estimated the standardized evolutionary response of phenotype, genotypes, and treatment effects in Haldanes (*h*) (see Supporting Information for the corresponding equations [equations 1 and 2]). Haldanes were used to estimate the evolutionary response as they are expressed in units of SD and a common scale allowed us to compare the magnitude of the genotypic and phenotypic responses (although we also report allele frequency differences). For both genotype and phenotype, the statistical significance of the mean selection intensity, mean evolutionary response, and treatment effects were determined using a *t*‐test with pond pairs as replicates. For the genotypic analysis, an individual's genotype was coded as a numeric trait (2 for two limnetic alleles, 1 for an individual with 1 limnetic and 1 benthic allele, 0 for two benthic alleles). We used linear models to describe the phenotypic trait trajectories through time. These models included a quadratic term that allowed us to model curvature in the trajectories through time. We quantified the difference between treatments within a family for both curvature and linear slope (equations 3 and 4 in the Supporting Information). We estimated standardized univariate selection differentials (intensities, *s’*) between sampling periods within a generation (i.e., September to January) as *s* *' = (* $\bar{x}$ *_after_ ‐* $\bar{x}$ *_before_)/* $\hat{\sigma }$ *_pooled_*. All statistical analyses were conducted in R (version 3.1.2; R Core Development Team 2018). All reported *P*‐values are two‐tailed.

## Results

### PHENOTYPIC TRAJECTORIES

**Figure 2:**
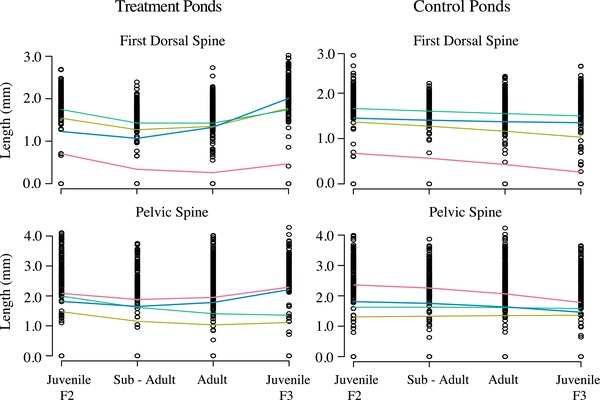
Trajectories of size corrected mean first dorsal spine and pelvic spine length through time in treatment and control ponds. Lines represent fitted values of quadratic regressions. Shared line color between panels identifies ponds within a pair (i.e., the same founding F_1_ family).

Trajectories of mean length of dorsal and pelvic spines in the experimental F_2_ generation populations diverged between treatments over time, and these differences were transmitted to the next (F_3_) generation (Fig. 2). Initially, over the first sampling interval, mean armor declined in all eight ponds, corresponding to the first summer and fall for the juvenile F_2_ generation stickleback (first dorsal spine, mean directional selection coefficients $\bar{s}$’ = −0.30 ± 0.07 SE, *t*_7_ = –4.24, *P* = 0.004; pelvic spine,$\;\bar{s}$’ = –0.15 ± 0.04 SE, *t*_7_ = –4.26, *P* = 0.004, treating ponds as independent replicates). Surprisingly, the initial decline in mean armor was significantly faster in ponds where trout were present than in control ponds (Fig. 2; statistical estimates of rate of change Table 1). This initial effect of treatment was found to be associated with reduced use of the open water habitat in the presence of trout, and increased use of the littoral zone (Rudman et al. 2016), where shorter spines are predicted to be favored (Reimchen 1994). Trajectories of mean dorsal and pelvic spine lengths began to reverse direction in the trout treatment ponds as the F_2_ cohort increased in body size over the winter and subsequent spring. This resulted in a significantly greater upward curvature of trajectories in both spine traits in ponds with trout predation (Fig. 2 and Table 1).

**Table 1:** Treatment effect on the linear slope and curvature of size corrected trait trajectories through time

	Treatment effect (95% CI)	*t*_3_	*P*‐value
First dorsal spine linear slope	−0.63 (−1.11 to 0.027)	−3.03	0.056
Pelvic spine linear slope	−0.73 (−1.22 to −0.24)	−4.73	0.018
First dorsal spine curvature	0.14 (0.002–0.277)	3.22	0.049
Pelvic spine curvature	0.15 (0.008–0.300)	3.37	0.043

### EVOLUTIONARY RESPONSE OF PHENOTYPE

**Figure 3:**
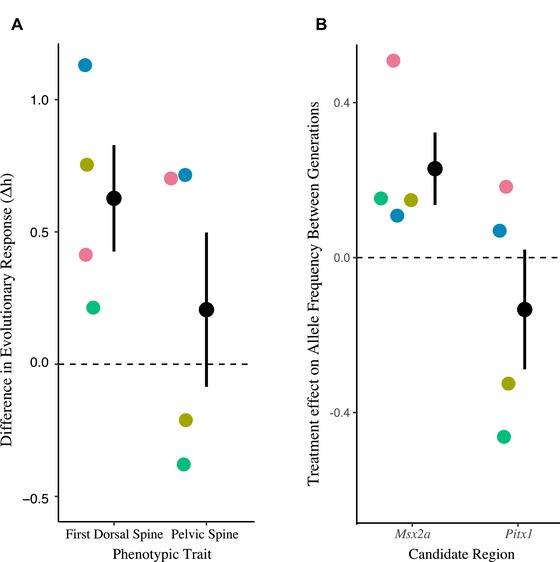
Evolutionary response of armor (A) and allele frequencies at two underlying genes (B). Dots above the line indicate more armor (longer spines or higher frequency of the limnetic alleles linked to longer spines) in the treatment ponds relative to control ponds. Black dots indicate overall mean with standard error. Individual colored dots represent pond pairs (F_1_ families).

After reproduction, mean length of first dorsal spine in the F_3_ cohort was greater in the treatment ponds than in control ponds, indicating an evolutionary response to vertebrate predation. In trout treatment ponds, mean first dorsal spine length in the next generation recovered from its initial decline to values similar to those of the F_2_ cohort at the start of the experiment, whereas the mean in the next generation declined in control ponds (Fig. 2). This resulted in divergent evolution of first dorsal spines between treatment and control ponds (mean treatment effect 0.63 $\bar{h}$ (haldanes) ±0.20 SE, *t*_3_ = 3.11, *P* = 0.052; Fig. 3A). Trends were the same in pelvic spine length, where treatment ponds showed a late‐life recovery from their initial decline, combined with weak selection on the trait in control ponds (Fig. 2). The net result after one pond generation was slight, but variable and nonsignificant, evolutionary divergence in pelvic spine length between treatment groups (0.21 $\bar{h}$ ± 0.29 SE, *t*_3_ = 0.71, *P* = 0.54; Fig. 3A).

### EVOLUTIONARY RESPONSE OF GENOTYPE

Our four F_1_ family QTL map (Fig. S6) indicated that length of the first dorsal spine maps to the region containing *Msx2a* on chromosome IV, and length of the pelvic spine and pelvic girdle map to the *Pitx1* region on chromosome VII, consistent with previous work (Chan et al. 2010; Howes et al. 2017). In the QTL maps within each F_1_ family peaks on chromosome IV near *Msx2a* explained an average of 9% of the variance (PVE) in first dorsal spine length and the peaks on chromosome VII near *Pitx1* explained on average 57% of the variance in pelvic spine length, depending on family (see Table S1 for individual F_1_ family values). Evolutionary changes in allele frequencies at the two major loci (*Msx2a* and *Pitx1*) underlying armor differences were commensurate with armor changes across the generations, confirming an evolutionary response at these genes. Alleles at *Msx2a* causing longer dorsal spines, inherited from the limnetic grandparents of the crosses, increased in frequency in treatment ponds relative to control ponds, with on average a 0.14 (±0.06 SE) difference in the frequency change of limnetic alleles. This allele frequency difference translated to an average standardized treatment effect of 0.23 $\bar{h}$ (±0.09 SE, *t*_3_ = 2.45, *P* = 0.09; a one‐tailed test based on the direction of phenotypic evolution is significant; Fig. 3B). Similar to the results on pelvic spine length, no significant treatment effect was detected at the *Pitx1* locus (–0.13 $\bar{h}$ ± 0.15 SE, *t*_3_ = –0.87, *P* = 0.45; Fig. 3B). The average difference in the change of limnetic allele frequency between predation and control ponds was –0.09 (±0.09 SE). *Pitx1* accounted for the majority of genetic variation in pelvic spine length in the F_2_ crosses (57% of variance on average), and the magnitude of the difference in allele frequency at this locus (Fig. 3A) was strongly correlated with the magnitude of the phenotypic difference in the trait between pond pairs (*r* = 0.99, *t*_2_ = 8.19, *P* = 0.015). In contrast, the genotype‐phenotype map for first dorsal spine is more complex, with *Msx2a* accounting for a smaller percentage of the variation in first dorsal spine length (9% variance on average among the four families). Accordingly, the magnitude of change in allele frequency was uncorrelated with the magnitude of the phenotypic shift between generations (*r* = −0.35, *t*_2_ = −0.68, *P* = 0.56)).

## Discussion

The phenotypic and ecological divergence of limnetic and benthic stickleback has been regarded as primarily a consequence of resource competition leading to differential foraging and habitat use (Schluter 1994). However, this differential habitat use has led to differential exposure to the community of predators. We show experimentally that spines and allele frequencies at the underlying genes evolved along different trajectories between trout addition and control ponds. This finding supports the hypothesis that divergence between sympatric stickleback is in part the outcome of their interactions with a vertebrate predator. We show that after a generation, an absence of vertebrate predators favors armor reduction, as has long been suspected (Nelson 1969; Reimchen 1980; Reimchen 1994). However, spine reduction was initially favored in both treatment and control ponds. The cause of this trend is not known but might have stemmed from differential mortality by insects, the main predators of juvenile stickleback, which has been hypothesized to select for reduced armor (Reimchen 1980; Reimchen 1994; Marchinko 2009). Early in life, armor reduction was favored even more strongly in the presence of the vertebrate predator than in its absence. In this experiment, this initial effect of treatment was shown to be linked to reduced use of the open water habitat and increased use of the littoral zone by individual fish in the presence of trout (Rudman et al. 2016), a behavioral response that may have heightened insect predation and selection in favor of shorter spines. Selection was later reversed in ponds with trout predators, favoring more armor (the ancestral marine phenotype). The large spatial scale of this experiment thus allowed behavioral responses to mediate the direction of selection, but it limited us to few replicates and hence manipulation of a single agent of biotic selection. Future experiments that manipulate multiple biotic agents, including insects, will be needed to disentangle the interactions between distinct predators and confirm our observed trajectories.

This experiment advances previous genetic mapping studies and transgenic experiments in stickleback (Chan et al. 2010; Howes et al. 2017), which identified genes contributing to variation in bony armor. Using artificial ponds, we manipulated a potential agent of selection on traits and key genes at a realistic biological scale. By measuring the evolutionary consequences of natural selection directly, we bypassed the need for fitness surrogates and strengthened the evidence for a heritable treatment effect. Thus, using a manipulative experiment, we provide one of the first examples in which the evolution of a phenotype has been linked to both the cause of selection and underlying genotype, which define critical steps in the modern study of the genetics of adaptation (Barrett and Hoekstra 2011; Barrett et al. 2019).

We also clearly attribute phenotypic and genotypic shifts to effects of a biotic interaction, in our case predation. Our results indicate that the ability to predict the evolutionary response at the genotypic level might depend on the complexity of the genotype‐phenotype map. The major effect of the *Pitx1* locus resulted in a much stronger correlation between the observed evolutionary responses at the level of phenotype and genotype than the minor effect *Msx2* locus. Aside from effect size, reduced predictability was likely also due to variation in epistatic effects among F_1_ families. Our relatively coarse scale mapping of the traits (due to the limited number of recombination events in an F_2_ cross) likely further contributed to reduced predictability. A caveat is that selection on linked genes and traits might also have contributed to treatment effects via correlated response. This is because *Msx2a* is located in a region of low recombination (Howes et al. 2017) also known to contain other genes affecting armor, body shape, and trophic traits (Albert et al. 2008; Howes et al. 2017). Future experiments are needed to disentangle individual genetic contributions to divergent evolution. Given the considerably larger effect size of *Pitx1* than *Msx2a* on the resultant phenotype, it is surprising that we observed a less consistent evolutionary response for pelvic spine length across replicates (i.e., increased spine length was disfavored in some families). Possible reasons for this variability include variable selection across replicates, differences in linkage disequilibrium between families, and sampling error. Although we do not explicitly examine competition its strength also likely varied between treatments. Stickleback density was temporally variable within the first generation and at the time of reproduction differed between the control and predation treatment ponds (Rudman et al. 2016); on average, there was a 65% reduction in the treatment pond populations compared to a 25% reduction in control ponds (Rudman et al. 2016). Interestingly population size reversed at the beginning of the F_3_ generation where on average treatment ponds had two times more fish than control ponds (Rudman et al. 2016).

Adaptive radiations are marked by explosions of new species having a diversity of ecological roles that often include herbivores, secondary consumers, and top predators (Schluter 2000; Seehausen 2006). Resource competition has been emphasized as the predominant biotic interaction driving these bursts. However, this view of biotic interactions in adaptive radiation does not explain divergence of sympatric, competing species in numerous traits not directly involved in resource acquisition (Thompson 1994; Jablonski 2008). It has also led to questions about whether the impact of biotic interactions in diversification are short‐lived and quickly wane over time, for example, as divergence proceeds and interspecific competition subsides (Hembry et al. 2014; Voje et al. 2015). Based on our findings, we suggest that evolving biotic interactions between any pair of diverging species can also lead to a cascade of changes in their interactions with other components of the food web in which they are embedded (Brodersen et al. 2018), in the present case accompanying differential habitat use, spurring further evolution. Thus, biotic interactions can sustain divergence in an ever expanding number of traits and genes, even in relatively low‐diversity environments such as postglacial lakes.

Associate Editor: Z. Gompert

## Supporting information


**Figure S1**. Aerial photograph of the experimental pond facility.
**Figure S2**. Experimental structure and timeline.
**Figure S3**. Relationship between standard length and spine length before and after size correction.
**Figure S4**. Variation in standard length across ponds and time.
**Figure S5**. Linkage Map.
**Figure S6**. QTL maps with F_1_ family as a covariate for the A) first dorsal and B) pelvic spine traits.
**Table S1**. Statistics associated with QTL mapping of first dorsal spine on chromosome IV near *Msx2a* and pelvic spine length on chromosome VII near *Pitx1* for each F_1_ family.
