# Supplementary material for: Genetics of adaptation: Experimental test of a biotic mechanism driving divergence in traits and genes
Source: Evol Lett. 2019 Aug 26;3(5):513–20. doi: 10.1002/evl3.135 (PMC6791182; doi:10.1002/evl3.135)
Supplement: Supplementary file 1 — Figure S1. Aerial photograph of the experimental pond facility. Figure S2. Experimental structure and timeline. Figure S3. Relationship between standard length and spine length before and after size correction. Figure S4. Variation in standard length across ponds and time. Figure S5. Linkage Map. Figure S6. QTL maps with F1 family as a covariate for the A) first dorsal and B) pelvic spine traits. Table S1. Statistics associated with QTL mapping of first dorsal spine on chromosome IV near Msx2a and pelvic spine length on chromosome VII near Pitx1 for each F1 family. [file EVL3-3-513-s001.pdf]

Online Supplement:

Supplementary Materials and Methods:

Phenotyping

Each fish was rehydrated over the course of a week in a series of ethanol - water washes (70%, 50%, 20%, 0% ethanol) and then fixed in 10% formalin. Specimens were stained with 0.001-0.002% w/v alizarin red S powder in a 2% w/v potassium hydroxide solution alizarin red to highlight bony structures, following the protocol outlined in Peichel et al., (1). Measurements were taken on the left side of each stained specimen. Fifty individuals per pond were measured in September 2012, January 2013, April 2013 and September 2013. We measured the length of the first dorsal spine, pelvic spine, and standard length. If a given trait was absent it was given a value of zero. Both spine traits scaled linearly with standard length (assessed using linear models), so trait values were size corrected to compare trait values among individuals of different sizes (see Figure S3 for an example of the relationship before and after correction). Traits were size corrected to an average length (32 mm, which was the mean size of individuals in September 2012 F_2 cohort; see Figure S4 for the size distributions across ponds and time points) using the equation: $Y_i = X_i - \beta(L_i - \bar{L})$, where Y_i is the size-adjusted armour trait, X_i is the original armour trait, β is the regression coefficient of the un-adjusted armour trait values on standard length, L_i is the standard length of the individual and $\bar{L}$ is the average length of the sample (2). Trait values of zero (*i.e.* when the trait was absent) were not corrected for size. When the trait values were plotted it was apparent that pelvic spine phenotypes fell into two distinct clusters, ‘high’ and ‘low’ armour. We used Gaussian Mixture Modelling for model-based clustering, using the mclust package (3), to identify these high and low pelvic phenotypes. These two pelvic armour clusters had different relationships (β) between standard length and the focal trait value; correspondingly size correction was done independently for the two clusters. Corrections were done

independently for each time period. For some time periods, linear models indicated that pond had a significant effect on β, in which case size correction was done independently for each pond. All analyses reported in this paper were undertaken using these size corrected measurements.

Library preparation, sequencing and genotyping

DNA was prepared for Illumina sequencing using the *PstI* enzyme following the genotyping by sequence method of Elshire *et al*. (4), with the addition of a gel size selection of fragments 500 – 700 base pairs (bp) in length. One hundred and ninety-two individuals were uniquely barcoded and combined into each library, for a total of seven libraries. Libraries were sequenced at the University of British Columbia's Biodiversity Next Generation Sequencing Centre on an Illumina HiSeq 2000. Reads were 100 bp in length and sequencing was paired end.

Sequence variants were identified using a standard, reference-based bioinformatics pipeline (see archived code for full details). After demultiplexing, Trimmomatic (5) was used to filter out low quality sequences and adapter contamination. Reads were then aligned to the stickleback reference genome (6) and a bacterial artificial chromosome (BAC) sequence containing the complete pituitary homeobox 1 (*Pitx1*) coding sequence (7) (GeneBank Accession: GU130435.1) using BWA v0.7.9a (8), subsequent realignment was done with STAMPY v1.0.23 (9). For genotyping the GATK v3.3.0 (10) best practices workflow (11) was followed except that the MarkDuplicates step was omitted. RealignTargetCreator and IndelRealigner were used to realign reads around indels and HaplotypeCaller identified single nucleotide polymorphisms (SNPs) in individuals. Joint genotyping was done across all individuals using GenotypeGVCFs. The results were written to a single VCF file containing all variable sites. This file was filtered for a minimum quality score (of 20) and depth of

coverage (minimum of 8 reads and maximum of 100,000) before use in any downstream analyses.

Linkage and quantitative trait locus (QTL) mapping

The pedigree of F_2 individuals was determined using the MasterBayes R package (12) using 1799 SNPs, which had minimal missing data across individuals (>90% of individuals with data). To have markers that were fully informative for linkage mapping we identified the SNPs that were homozygous for alternative alleles in the benthic and limnetic grandparents of each F_2 cross. We then used these SNPs to calculate pairwise recombination frequencies and create a genetic map using JoinMap version 3.0 (13). In total 398 F_2 progeny from four F_1 crosses were used for mapping, comprised of multiple F_2 families. F_2 genotypes were coded according to the population code for outbred crosses, allowing segregation of up to four alleles per locus (cross-pollinator). The JMGRP module of JoinMap was used with a LOD score threshold of 4.0 to assign 2243 loci to 33 linkage groups. For each linkage group, a map was created with the JMMAP module (Figure S5). Mapping was done using the Kosambi function with a LOD threshold of 1.0, recombination threshold of 0.499, jump threshold of 5.0, and no fixed order. Two rounds of mapping were performed, with a ripple performed after each marker was added to the map.

A total of 2243 SNP markers and the genetic map were used for the quantitative trait locus (QTL) mapping of first dorsal spine and pelvic spine length (Figure S6). QTL mapping was done using the Haley–Knott regression with F_1 family as a covariate in the R/qtl package (14). To test whether there was variation among families in the genetic basis of each trait the QTL mapping was also done independently for each family, with a sample size of 99-100 individuals per family. Percentage variance explained (PVE) for each trait was calculated using the following equation:

PVE = 1 - (10^(-2*(LOD/ n))),

where LOD is the estimated LOD score and n is the sample size.

Genotypic estimates of selection were averaged across all SNPs that fell between the QTL peaks identified for first dorsal spine and pelvic spine phenotypes and the coding regions of the candidate genes (*Msx2a* and *Pitx1* respectively). The region between the all F_1 family QTL peak and the coding regions of the genes spanned 0.21 cM or 1,661 kb for *Msx2a* and 3 cM or 2,886 kb for *Pitx1*. For selection to be estimated SNPs were required to have data for at least 30 individuals per pond per time point. Correspondingly, the number of informative SNPs varied between families and traits (7-13 SNPs for the *Msx2a* locus and 5-6 SNPs for the *Pitx1* locus). Selection was estimated for each SNP independently, then the individual estimates of selection were averaged across the SNPs that were informative in each family.

Selection Analyses

We estimate the standardized evolutionary response of phenotype in Haldanes (h) as:

Equation 1: $h = \frac{\Delta \bar{Z}}{g}$,

where $\Delta \bar{Z}$ is evolutionary response,

$\Delta \bar{Z} = (\bar{z}_{\text{after}} - \bar{z}_{\text{before}})/\hat{\sigma}_{\text{pooled}}$,

$\bar{z}_{\text{before}}$ and $\bar{z}_{\text{after}}$ are the mean phenotype or allele frequency in the generation before and after selection, $\hat{\sigma}_{\text{pooled}}$ is the square root of pooled sample variance of the trait or allele frequency in the first and second generation, and g is the number of generations of selection, which in our case is 1 (15).

We estimate the treatment effect of evolutionary response within a pond pair (Δh) as:

Equation 2: $\Delta h = h_t - h_c$

where h_t is the evolutionary response in the trout addition pond and h_c is the evolutionary response in the control pond.

We used the same formulas and methods to estimate the evolutionary response and treatment effect for allele frequencies at SNP markers near the QTLs for pelvic spine and first dorsal spine length. The input was the mean limnetic allele frequency for the individuals in the sample, standard deviation was also estimated from these allele frequencies.

We used linear models to describe the phenotypic trait trajectories through time. These models included a quadratic term which allowed us to model curvature in the trajectories through time. We quantified the difference between treatments within a family for both curvature and linear slope as follows:

Equation 3: $\Delta slope = slope_t - slope_c$

Equation 4: $\Delta curvature = curvature_t - curvature_c$

where $slope_t$ or $curvature_t$ is the variable in the trout addition pond and $slope_c$ or $curvature_c$ is the variable in the control pond.

References

1. Peichel CL, Nereng KS, Ohgi KA, Cole BL, Colosimo PF, Buerkle CA, Schluter D, Kingsley DM (2001) The genetic architecture of divergence between threespine stickleback species. *Nature* 414:901–905.
2. Vamosi SM (2002) Predation sharpens the adaptive peaks: survival trade-offs in sympatric sticklebacks. *Ann. Zool. Fennici* 39:237–248.
3. Fraley C, Raftery AE (2012) Package 'mclust'. Normal mixture modeling for model-based clustering, classification, and density estimation (Tech. Rep. 597, University of Washington, United States).
4. Elshire RJ, Glaubitz JC, Sun Q, Poland JA, Kawamoto K, Buckler ES, Mitchell SE (2011) A robust, simple genotyping-by-sequencing (GBS) approach for high diversity species. *PloS one* 6:e19379.
5. Bolger AM, Lohse M, Usadel B (2014) Trimmomatic: a flexible trimmer for Illumina sequence data. *Bioinformatics* 30:2114–2120.
6. Jones FC, Garbherr MG, Chan YF, Russel P, Mauceli E, Johnson J, Swofford R, Pirun M, Zody MC, White S, Birney E, Searle S, Schmutz J, Grimwood J, Dickson MC, Myers RM, Miller CT, Summer BR, Knecht AK, Brady AD, Zhang H, Pollen AA, Howes T, Amemiya C, Broad Institute Genome Sequencing Platform and Whole Genome Assembly Team, Lander ES, Di Palma F, Lindblad-Toh K, Kingsley DM (2012) The genomic basis of adaptive evolution in threespine sticklebacks. *Nature* 484:55–61.
7. Chan YF, Marks ME, Jones FC, Villarreal G, Shapiro MD, Brady SD, Southwick AM, Absher DM, Grimwood J, Schmutz J, Myers RM, Petrov D, Jonsson B, Schluter

D, Bell MA, Kingsley DM (2010) Adaptive evolution of pelvic reduction in sticklebacks by recurrent deletion of a *Pitx1* enhancer. *Science* 327:302–305.

8. Li H, Durbin R (2009) Fast and accurate short read alignment with Burrows–Wheeler transform. *Bioinformatics* 25:1754–1760.
9. Lunter G, Goodson M (2011) STAMPY: a statistical algorithm for sensitive and fast mapping of Illumina sequence reads. *Genome Res.* 21:936–939.
10. McKenna A, Hanna M, Banks E, Sivachenko A, Cibulskis K, Kernytsky A, Garimella K, Altshuler D, Gabriel S, Daly M, DePristo MA (2010) The Genome Analysis Toolkit: a MapReduce framework for analyzing next-generation DNA sequencing data. *Genome Res.* 20:1297–1303.
11. DePristo MA, Banks E, Poplin R, Garimella KV, Maguire JR, Hartl C, Philippakis AA, Del Angel G, Rivas MA, Hanna M, McKenna A (2011) A framework for variation discovery and genotyping using next-generation DNA sequencing data. *Nature Genetics* 43:491–498.
12. Hadfield J (2012) Masterbayes: ML and MCMC methods for pedigree reconstruction and analysis. CRAN: Comprehensive R Archive Network. Available at: http://www.r-project.org.
13. Ooijen JW, Voorrips RE (2002) JoinMap: Version 3.0: Software for the Calculation of Genetic Linkage Maps, Plant Research International. Wageningen, The Netherlands.
14. Broman KW, Wu H (2013) QTL: Tools for Analyzing QTL Experiments. CRAN: Comprehensive R Archive Network. Available at: http://www.r-project.org.
15. Hendry AP, Kinnison MT (1999) Perspective: the pace of modern life: measuring rates of contemporary microevolution. *Evolution* 53:1637–1653.

Supplementary Figure 1. Aerial photograph of the experimental pond facility. Length and width of the ponds are indicated in the photo. The authors of the study are also picture adjacent to the pond for reference of scale. Photo courtesy of Thor Veen.

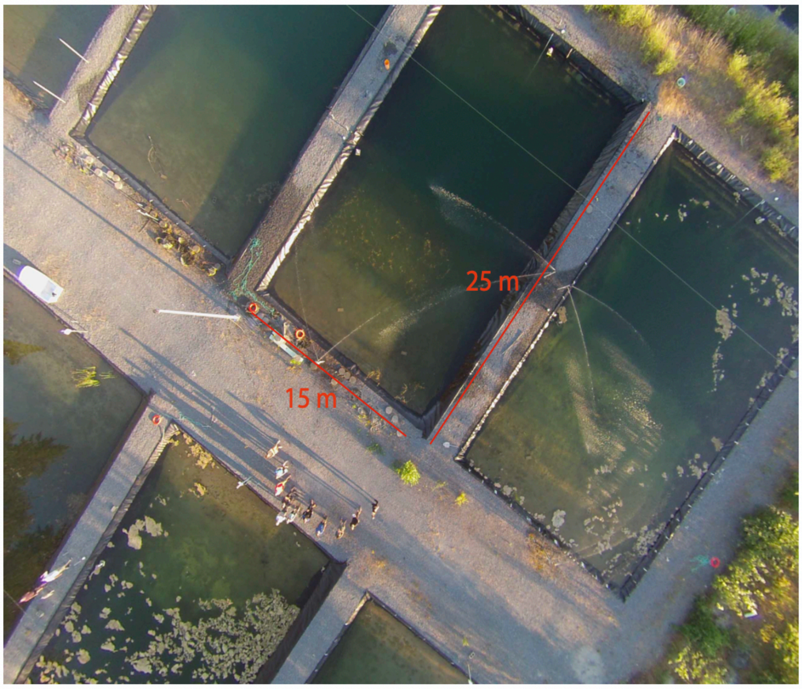

Supplementary Figure 2. Experimental structure and timeline. Shared color between ponds indicates the same founding F_1 family.

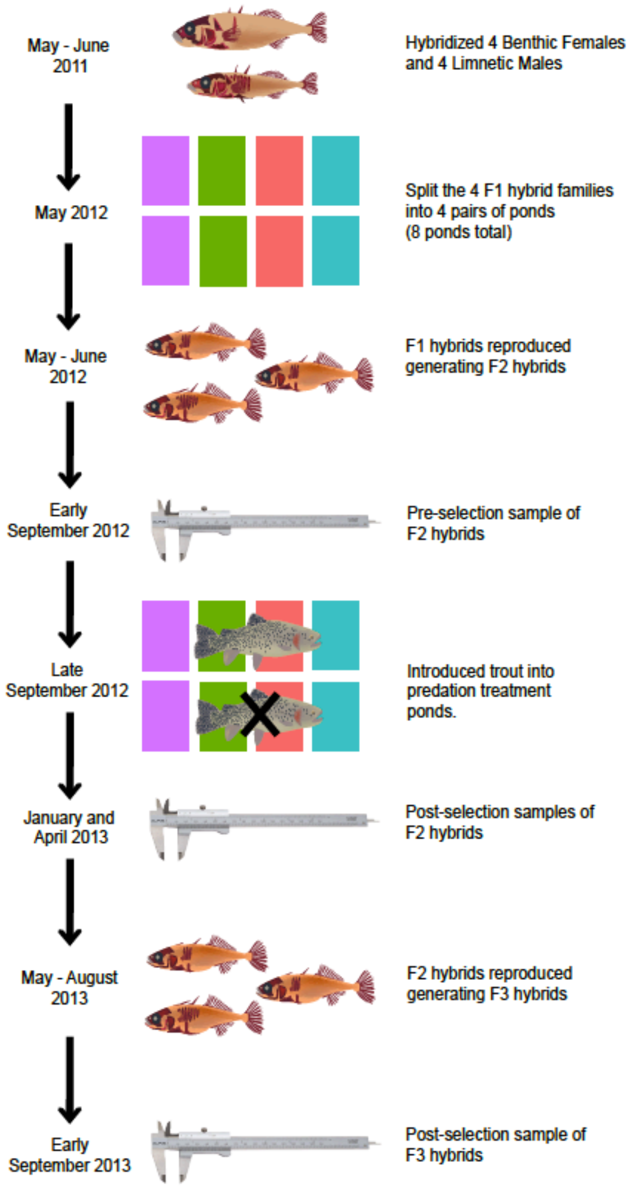

Supplementary Figure 3. Relationship between standard length and spine length before and after size correction. Plotted data are from all ponds, the four sampling periods are indicated by colour.

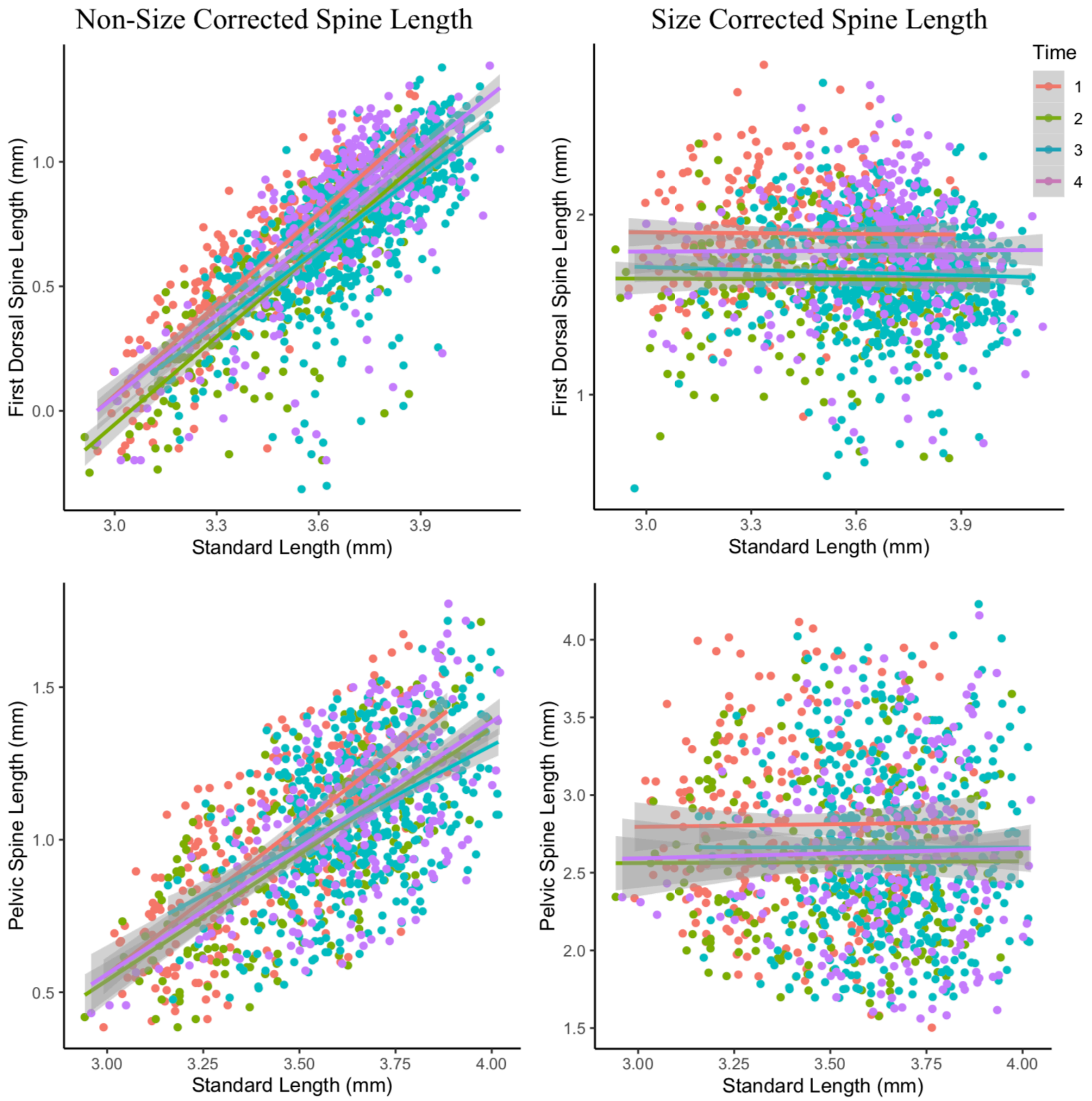

Supplementary Figure 4. Variation in standard length across ponds and time.

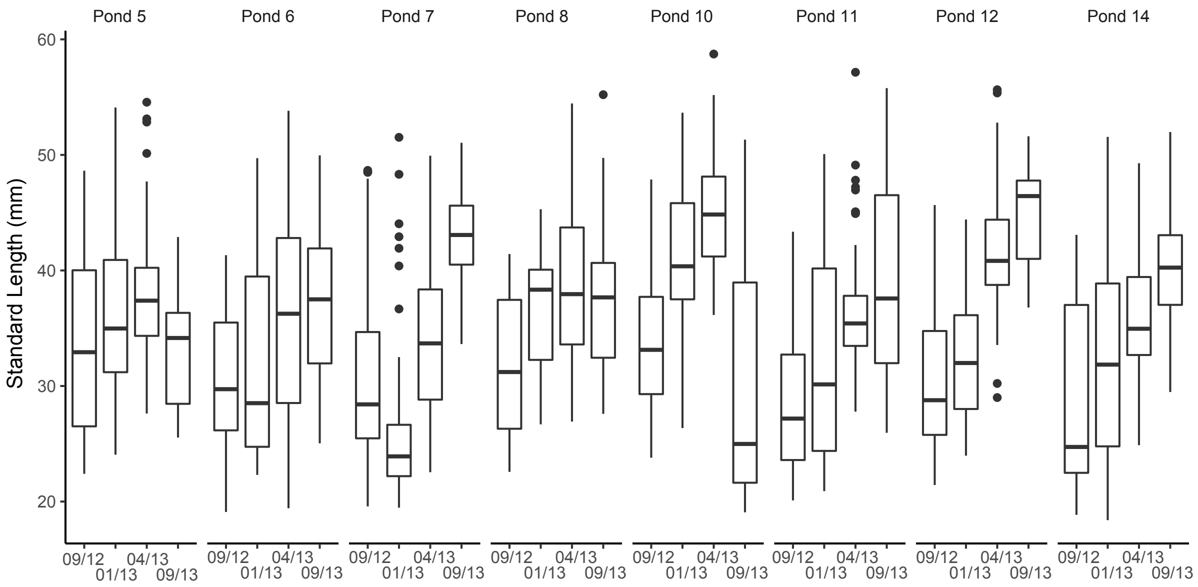

Supplementary Figure 5 Linkage Map.

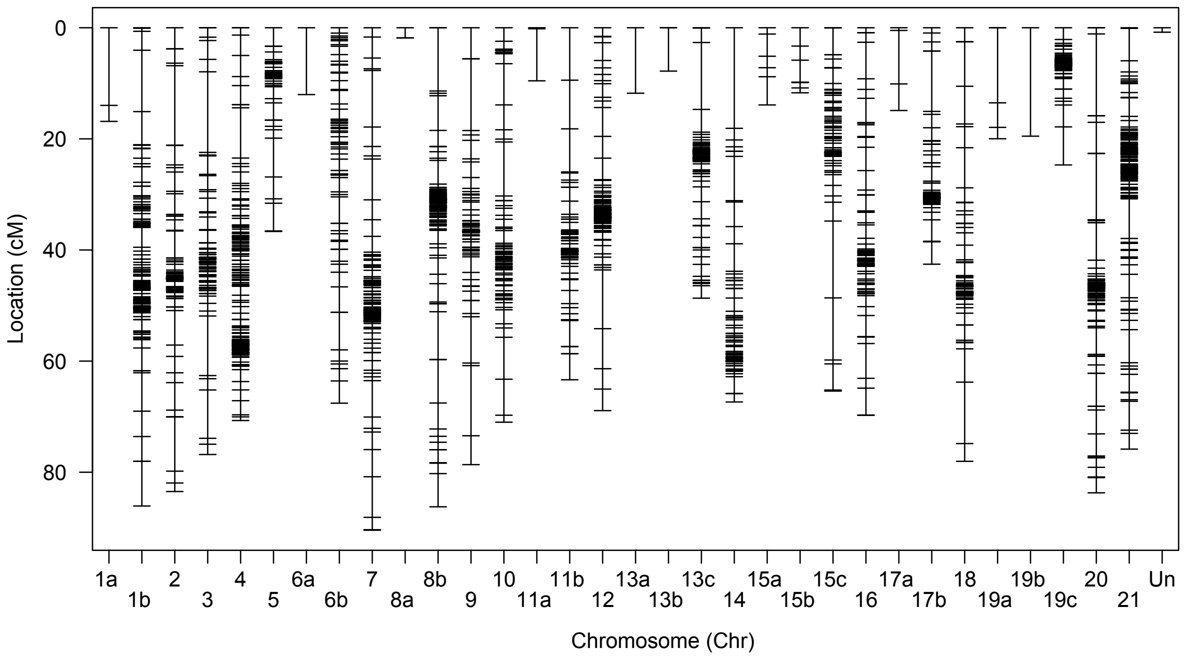

Supplementary Figure 6. QTL maps with F_1 family as a covariate for the A) first dorsal and B) pelvic spine traits. The location of the candidate genes for each trait are indicated in red.

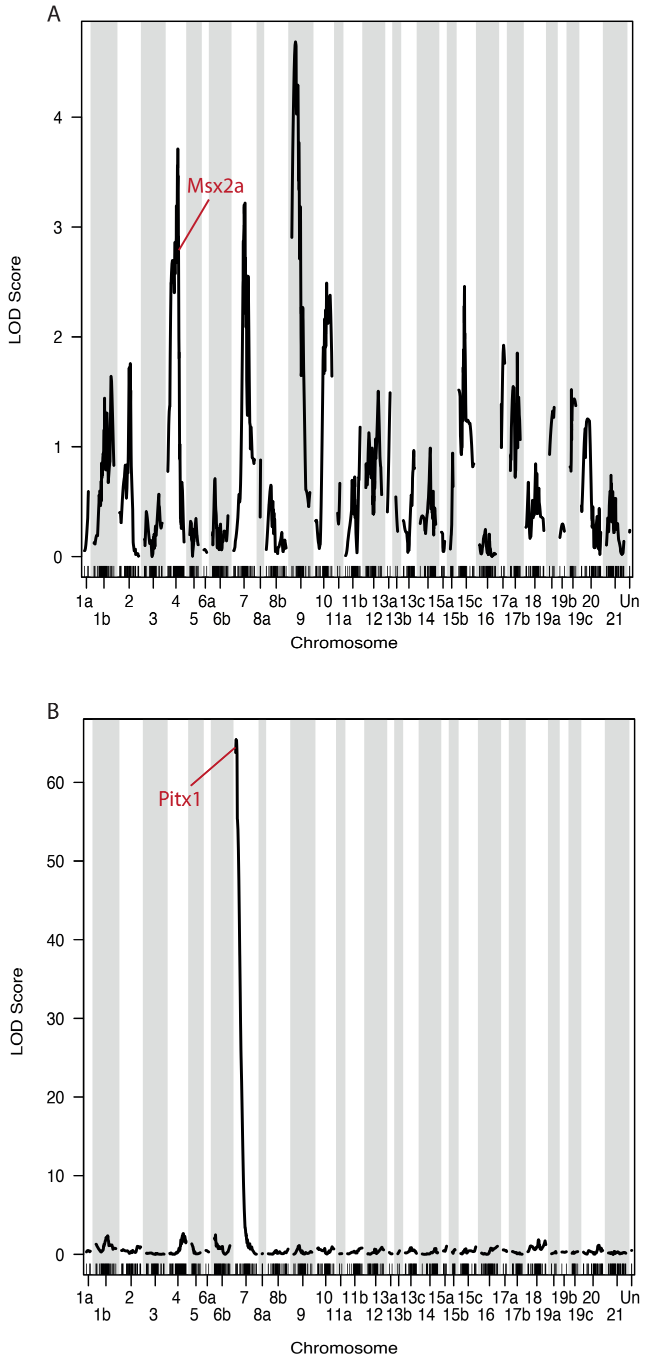

Supplementary Table 1. Statistics associated with QTL mapping of first dorsal spine on chromosome IV near *Msx2a* and pelvic spine length on chromosome VII near *Pitx1* for each F_1 family.

F_1 Family	LOD Score First Dorsal Spine	PVE First Dorsal Spine	LOD Score Pelvic Spine	PVE Pelvic Spine
1	1.69	8.0	21.49	63.0
2	2.00	9.0	17.97	56.0
3	0.69	3.0	15.33	51.0
4	3.89	16.0	18.50	57.0
